# Saturated fatty acids inhibit unsaturated fatty acid induced glucose uptake involving GLUT10 and aerobic glycolysis in bovine granulosa cells

**DOI:** 10.1038/s41598-024-59883-x

**Published:** 2024-04-30

**Authors:** Xuelian Tao, Maryam Rahimi, Marten Michaelis, Solvig Görs, Julia Brenmoehl, Jens Vanselow, Vijay Simha Baddela

**Affiliations:** 1 Research Institute for Farm Animal Biology (FBN), Wilhelm-Stahl-Allee 2, 18196 Dummerstorf, Germany; 2 Abteilung Biotechnologie und Reproduktion Landwirtschaftlicher Nutztiere, Georg-August-Universität Göttingen, 37037 Göttingen, Germany

**Keywords:** Fatty acids, Glucose, Metabolism, Granulosa cells, GLUT10, Cell biology, Physiology, Endocrinology

## Abstract

Fatty acids have been shown to modulate glucose metabolism in vitro and in vivo. However, there is still a need for substantial evidence and mechanistic understanding in many cell types whether both saturated and unsaturated fatty acids (SFAs and UFAs) pose a similar effect and, if not, what determines the net effect of fatty acid mixes on glucose metabolism. In the present study, we asked these questions by treating granulosa cells (GCs) with the most abundant non-esterified fatty acid species in bovine follicular fluid. Results revealed that oleic and alpha-linolenic acids (UFAs) significantly increased glucose consumption compared to palmitic and stearic acids (SFAs). A significant increase in lactate production, extracellular acidification rate, and decreased mitochondrial activity indicate glucose channeling through aerobic glycolysis in UFA treated GCs. We show that insulin independent glucose transporter GLUT10 is essential for UFA driven glucose consumption, and the induction of AKT and ERK signaling pathways necessary for GLUT10 expression. To mimic the physiological conditions, we co-treated GCs with mixes of SFAs and UFAs. Interestingly, co-treatments abolished the UFA induced glucose uptake and metabolism by inhibiting AKT and ERK phosphorylation and GLUT10 expression. These data suggest that the net effect of fatty acid induced glucose uptake in GCs is determined by SFAs under physiological conditions.

## Introduction

Oleate (OA, C18:1), palmitate (PA, C16:0), and stearate (SA, C18:0) are the predominant long-chain fatty acids in adipose tissue, constituting up to 70% of esterified fatty acid fraction, plausibly due to their abundant concentration in the human/animal diet and their synthesis by the body’s own metabolism^1,2^. These three fatty acids are also the most prevalent non-esterified fatty acids (NEFA) in circulation and other body fluids, including ovarian follicular fluid^3,4^. Increased lipid mobilization in diabetes and negative energy balance, and lipid overflow during obesity lead to elevated levels of non-esterified OA, PA, and SA in the circulation^5^. NEFAs, also known as free fatty acids (FFAs), are released from the hydrolysis of triglycerides in adipose tissue. NEFAs in blood will be taken up by tissues to meet the local energy demands, while excess amounts of NEFAs will be stored in lipid droplets in different tissues, which could cause or contribute to various disorders such as steatosis, insulin resistance, and infertility^5–9^.

In humans and animals, consumption of lipid-rich foods has been shown to affect glucose homeostasis^10,11^. Intake of a diet rich in PA was found to reduce the β-cell function and insulin sensitivity, leading to adverse effects on postprandial glucose metabolism. In contrast, intake of OA-rich food progressively improves insulin sensitivity and β-cell function in humans^12,13^. Similarly, dietary alpha linolenic acid (ALA, C18:3) supplementation was found to improve glucose tolerance, reduce insulin resistance, and decrease hepatic steatosis^14,15^. Supplementation of PA prevented the insulin gene transcription in β cells and caused insulin resistance in primary mouse hepatic cells in vitro^16,17^. Therefore, elevated levels of saturated fatty acids (SFAs) were implied to be an important causative agents for insulin resistance where as unsaturated fatty acids (UFAs) were recognized for the health beneficial effects. It is well known that glucose transport into the cells is facilitated by glucose transporter proteins (GLUTs). Fourteen different GLUTs have been identified with different kinetic properties, substrate selectivity, and specific distribution patterns^18,19^. GLUT1 and GLUT3 are expressed in the brain, mammary epithelial cells, placenta, and fetal tissues. GLUT 1 is also reported to be expressed in ovarian follicular cells^20–22^. Because of its ubiquitous expression and high affinity to glucose, GLUT1 is considered to be responsible for basal glucose uptake in the body. GLUT2 has a low affinity to glucose and is expressed in hepatocytes and pancreatic beta cells. GLUT 4 is the insulin-dependent glucose transporter that facilitates glucose uptake in adipose tissue and skeletal and cardiac muscles^23,24^. Upon uptake by a cell, glucose is channeled through the glycolytic pathway for energy purposes and yields either pyruvate or lactate, depending on the energy metabolism^25–27^.

Ovarian follicles are the functional units of an ovary and contain the germ cell oocyte surrounded by layers of steroidogenic granulosa cells (GCs) producing sex steroid estradiol. An increasing number of publications suggest that glycolytic energy metabolism is essential for ovarian follicle function. Zhang et al. (2022) showed that enhanced glycolysis in GCs activates primordial follicles to be recruited into folliculogenesis in mice^28^. It was found that glucose can be converted to lactate by in-vitro cultured mouse pre-antral follicles at concentrations ranging from 1 to 5 mM of glucose^29^. Gonadotropin supplementation could further induce lactate accumulation in cultured murine ovarian antral follicles in vitro^30^. Also, in sheep, glycolytic metabolism was upregulated in gonadotropin-stimulated antral follicles, as reflected by increased glucose uptake and lactate production^31^. Abigail et al. found that GCs of small follicles exhibit enhanced glucose uptake and glycolytic metabolism in bovine^32^. In vitro studies in pigs showed that increased proliferation of GCs in small follicles undergoing development is accompanied by a metabolic shift toward aerobic glycolysis^33^, indicating that the glycolytic pathway is a major player of GCs function in different species.

Elevated levels of NEFAs have been shown to impair GC function^34–36^. Although it was found that PA inhibits glucose uptake in human granulosa-like tumor cells (KGN)^37^, it is unclear how different fatty acids affect glucose metabolism in primary GCs. Therefore, the present study was conducted to understand the effects of SFAs (PA and SA) and UFAs (OA and ALA) individually and in physiological mixes on glucose consumption and cellular metabolism in primary bovine GCs using our in vitro culture model^38^.

## Materials and methods

### Collection of ovaries, isolation of GCs, and in-vitro culture

Primary GCs isolation, cryopreservation, and culture were performed according to our previously published method^38^. Briefly, bovine ovaries were obtained from the DANISH CROWN Teterower Fleisch GmbH, Germany, and transported to the laboratory in sterile saline solution. GCs were aspirated from ≤ 6 mm sized ovarian follicles using a 3 ml syringe and 18-gauge needles. Viable cells were counted via the Trypan Blue staining exclusion method and cryopreserved in freezing medium (90% Fetal Calf Serum and 10% DMSO). It was reported that GCs preparation may constitute 2–6% of contaminant cell types^39,40^. For cell culture, GCs were seeded at a density of 5 × 10^4^ cells/well in 48-well culture dishes, which were pre-coated with 0.02% Collagen R (Serva, Germany) to promote attachment. Cells were cultured in a growth medium (α-minimum essential medium containing 2 mM glutamine, 10 mM sodium bicarbonate, 0.1% w/v BSA, 20 mM HEPES, 4 ng/ml sodium selenite, 5 µg/ml transferrin, 10 ng/ml insulin, 1 mM non-essential amino acids, 100 IU/ml penicillin and 0.1 mg/ml streptomycin) supplemented with follicle stimulating hormone (FSH; 20 ng/ml, Sigma-Aldrich), IGF1 (R3-IGF1, 50 ng/ml, Sigma-Aldrich) and Androstenedione (2 mM, Sigma-Aldrich). All cell cultures were maintained at 37 °C and 5% CO_2_ in a humidified incubator. Fatty acids were added to the cultured GCs on days 2 and 4 by replacing 70% of the spent medium with a fresh medium containing fatty acids. On day 6, the spent media were collected to assay glucose and lactate levels, and cells were lysed for RNA and protein expression analysis. Brightfield photomicrographs of cells with different fatty acid treatments were presented in Supplementary Fig. 1 and an outline of all analyses employed in the study was presented in Supplementary Fig. 2.

To investigate Akt and ERK signaling, GCs were treated with 50 μM of corresponding signaling inhibitors LY294002 (Cell signaling technology, USA) and PD98059 (Cell signaling technology, USA) on day 2. After 2 h of inhibitor treatment, the medium was replaced with fresh medium containing fatty acids and the culture continued until day 4. For SLC2A10 knockdown, cells were treated with 25 nM of antisense LNA negative control GapmeR (AACACGTCTATACGC) and antisense SLC2A10 GapmeR oligos (CAGTGCATAGTTGAGC) on day 2, using the TransIT-X2® transfection reagent (Mirus Bio, USA) as per the manufacturer’s recommendations. The spent medium was replaced with fresh medium containing fatty acids on day 3 and 5, and cells were analyzed on day 7.

### Fatty acids preparation

All fatty acids were prepared by dissolving in 10% fatty acid-free bovine serum albumin (BSA) solution to a 5 mM stock solution containing a fatty acid to BSA molar ratio of 3.3. Since SFAs are barely directly soluble in the BSA solution, they were first dissolved in a chloroform/methanol solution (v/v = 2/3) and dried under a nitrogen gas chamber. The fatty acid solutions were vortexed, sonicated and then placed in a water bath at 37 °C overnight to form a clear solution. The solution was then filtered through a 0.22 µm filter and stored at − 20 °C for cell culture usage in aliquots.

### Glucose and lactate measurement

The spent media at the end of the culture was collected in 1.5 ml tubes, and centrifuged at 3000×*g* for 5 min. Cell-free spent media was then transferred to a fresh 1.5 ml tube ready for measurement. Glucose and lactate concentration were measured by an automatic enzymatic analyzer (ABX Pentra 400, HORIBA Medical, Montpellier, France) with the commercial kits (for glucose: Kit No. A11A01667, for lactate: Kit No. A11A01721, Axon Lab AG). The inter-assay coefficients of variation (CV) for glucose and lactate were 0.9% and 0.6%, respectively.

### Extracellular acidification rate (ECAR) analysis

ECAR was analyzed using a seahorse XFe96 Analyzer (Agilent Technologies, Santa Clara, CA, USA) and XF glycolysis stress test kit (Kit No.103020-100, Agilent Technologies) according to the manufactururs instructions. GCs were seeded in a 96-well Seahorse XF cell culture plate at a density of 2.0 × 10^4^ cells per well and cultivated in growth media for six days. Fatty acids or BSA supplementation was administered with fresh medium on days 2 and 4. Cells were analyzed on day 6 as in the other tests. Before starting the Seahorse measurements, cells in microplates were washed twice with glycolysis stress test assay medium containing 2 mM l-Glutamine (starvation media) and then incubated in the same medium at 37 °C for 1 h. Meanwhile, glucose at a final concentration of 10 mM, oligomycin at a final concentration of 2 μM, and 2-deoxy-glucose (2DG) at a final concentration of 50 mM were loaded into the appropriate ports of a seahorse sensor cartridge that had been hydrated with seahorse XF Calibrant solution on the previous day. During the experiment, these reagents were automatically added to the cells one by one to pharmacologically stimulate or inhibit the glycolysis and mitochondrial electron transport (glucose: glycolysis stimulant, oligomycin: mitochondrial ATP synthase complex inhibitor, 2DG: a hexokinase and glycolysis inhibitor) for analyzing the ECAR. After completing the assay, the medium was discarded and Hoechst 33342 reagent [(8.9 µM in DMEM) B2261, Sigma-Aldrich] was added and incubated for 10 min at 37 °C to determine the cell amount per well by using a plate reader (FLUOstar Omega, BMG Labtech). The Hoechst measurements were used to normalize Seahorse data. Glycolytic functions were determined from ECAR data using Test Report Generators (Agilent).

### 2-NBDG glucose uptake assay

**Figure 1:**
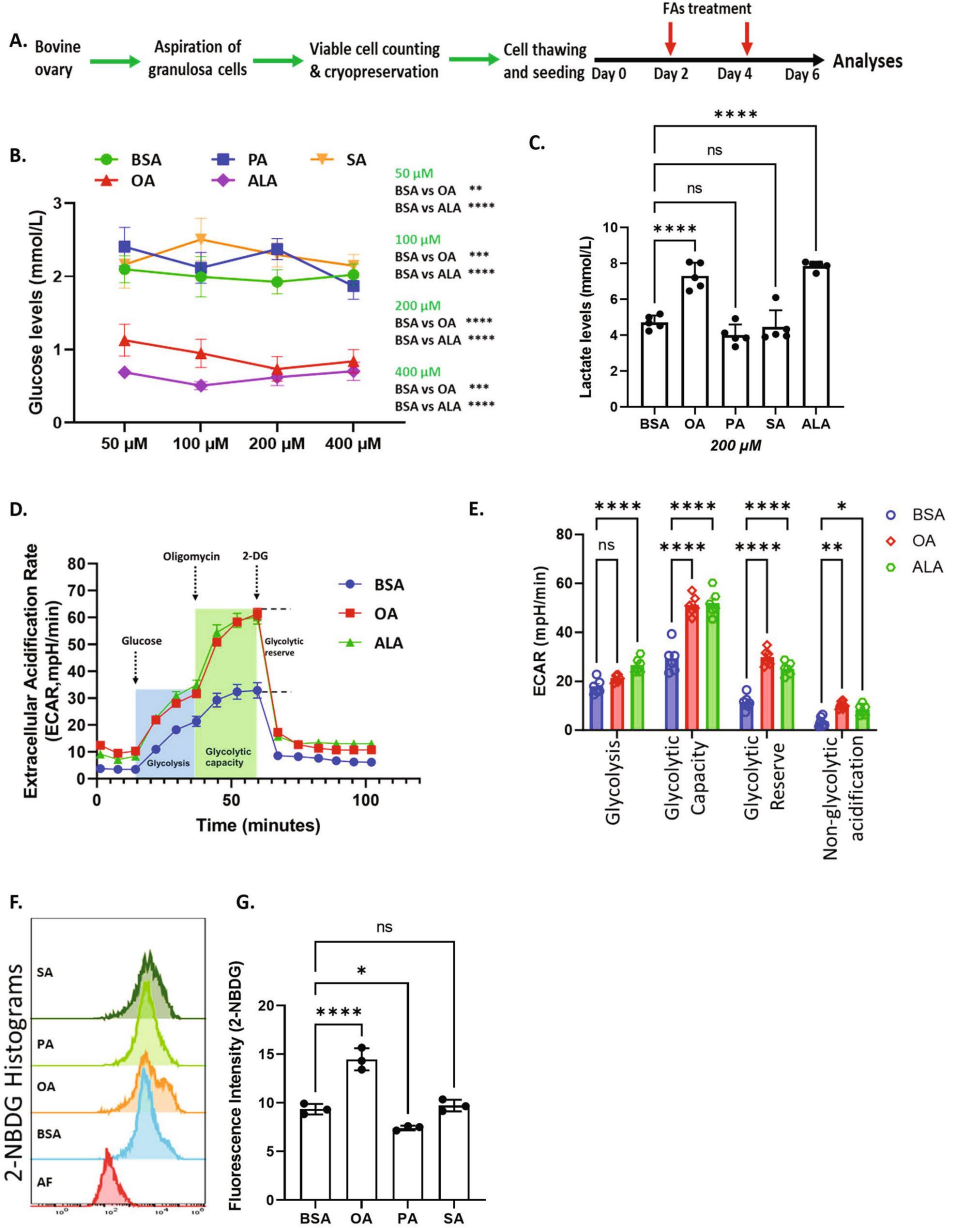
UFAs induce glucose uptake and lactate production. (**A**) Schematic representation of cell culture and treatment procedure for all analyses. (**B**) Glucose level in the spent media of GCs cultured under different NEFA treatments at different concentrations (n = 7). Bovine serum albumin, oleate, palmitate, stearate and alpha-linolenic acid was abbreviated as BSA, OA, PA, SA and ALA. (**C**) Lactate levels in the spent media of GCs (n = 5). (**D**) Glycolysis reflected by the extracellular acidification rate (ECAR) measured in GCs treated with 200 µM of OA, ALA, and BSA control. (n = 6). (**E**) Quantification of different glycolytic functions such as glycolysis, glycolytic capacity, glycolytic reserve and non-glycolytic acidification from ECAR data (n = 6). (**F**) Representative flow cytometry histograms generated in 2-NBDG analysis. “AF” represents autofluorescence. (**G**) 2-NBDG uptake in GCs treated with different fatty acids at 200 µM concentration (n = 3, each n is a pool of two independent culture replicates). Probability values < 0.05 were considered statistically significant and are designated with up to four asterisk symbols to inform the strength of significant difference (*p < 0.05; **p < 0.01; ***p < 0.001, ****p < 0.0001). n = indicates the number of independent cell culture replicates analyzed.

2-NBDG (N13195, ThermoFisher Scientific, Germany) is a fluorescent glucose analog that is taken up by living cells and allows monitoring of glucose uptake. GCs were plated in 48-well plates with 5 × 10^4^ cells/well and treated with BSA, PA, SA and OA as outlined in Fig. 1A. On day 6, cells were equilibrated in a glucose-free medium for 1 h followed by incubation with 200 µl of ready-to-use Accutase solution (A6964, Sigma-Aldrich, Germany) for 20 min at 37 °C to detach cells. Cells were transfered into 1.5 ml tubes and washed twice with glucose-free media by centrifugation at 300×*g* for 5 min. Cells were then incubated with 2-NBDG at a final concentration of 400 µM for 60 min at 37 °C. Prior to analysis by flow cytometry (Gallios, Beckman-Coulter), propidium iodide (PI) was added to the cells at a final concentration of 5 µg/ml to mark dead cells. During the flow analysis, cells were excited by a 488 nm laser and the emission were recorded at 525 ± 25 nmfor 2-NBDG and 620 ± 30 nm for PI. The fluorescence signal intensities were analyzed using the Kaluza 1.2 software (www.beckman.de). Forward versus side scattering gating was employed to analyze the cell morphology and exclude the debris from the analysis where as fluorescence signals from PI staining were used to exclude the dead cells from the analysis.

### JC1 staining for mitochondrial membrane potential analysis

GCs were plated in 48-well plates with 5 × 10^4^ cells/well and treated with fatty acids as mentioned earlier. Culture media was removed from the plate and cells were washed twice with 37 °C pre-warmed DMEM. Cells were then incubated with 2 µM JC-1 reagent (T3168, ThermoFisher Scientific, Germany) in DMEM for 30 min at 37 °C. Under low mitochondrial membrane potential (MMP), JC-1 maintains its monomeric form and emits green fluorescence. On the contrary, JC-1 monomers aggregate and emit red fluorescence at high MMP. After staining, cells were washed once in DMEM and subjected to endpoint measurements using a fluorescence plate reader (FLUOstar Omega, BMG Labtech), with an excitation wavelength at 485 ± 10 nm (for both aggregate and monomer) and emission wavelengths at 590 ± 10 nm (for aggregate only) and 510 ± 10 nm (monomers only). To verify the effectiveness of the concentration of JC-1, GCs treated with or without 2 µM valinomycin were initially analyzed in a flow cytometer (Gallios, Beckman-Coulter, Germany) using the same excitation and emission wavelengths mentioned above. Detachment of cells and signal quantification was performed as described for 2NBDG analysis.

### Fluorescence microscopy

Granulosa cells were cultured in Nunc™ Lab-Tek™ Chamber Slides for immunofluorescence imaging. Cell culture media was removed and cells were washed twice with 37 °C pre-warmed DMEM. Cells were then incubated with BioTracker 488 Green Lipid Droplet Dye (1:400 in DMEM; SCT144, Sigma-Aldrich) for 30 min at 37 °C. After washing twice with DMEM, cells were incubated with MitoTracker™ Orange CMTMRos (300 nM in DMEM; M7510, ThermoFisher Scientific) for 30 min at 37 °C. Cells were washed again and incubated with Hoechst 33342 (8.9 µM in DMEM; B2261, Sigma-Aldrich] for 10 min at 37 °C. After a final wash step in DMEM, cells were analyzed using a Carl Zeiss confocal laser scanning microscope LSM 800 with a 40 × oil lens (magnification 400x). Fluorescence signal was acquired by excitation of each dye with specific laser light (405 nm for Hoechst, 488 nm for Lipid Droplet Dye and 561 nm for MitotrackerOrange). Brightfield images were generated using the ESID (Electronically switchable illumination and detection module) detector with a 640 nm laser.

### ATP production measurement

Cellular ATP quantification was performed using the BioTracker™ ATP-Red Live Cell Dye (SCT045, Sigma-Aldrich, Germany). Cultured GCs were detached by incubation with 200 µl of ready-to-use Accutase (A6964, Sigma-Aldrich, Germany) for 20 min at 37 °C. Cells were collected by centrifugation at 300×*g* for 5 min into 1.5 ml tubes, and washed twice with 37 °C pre-warmed DMEM. Cells were incubated with BioTracker ATP-Red Live Cell Dye (1:400 in DMEM) for 30 min at 37 °C. Cells were washed again and PI was added to the cells at a final concentration of 5 µg/ml to mark dead cells. The fluorescence signal of single cells (10,000 counts) was quantified by flow cytometry (Gallios, Beckman-Coulter) and the data were analyzed with Kaluza 1.2 software (www.beckman.de).

### RNA extraction, cDNA synthesis and qPCR

Total RNA from GCs was extracted using the innuPREP RNA Mini Kit (Analytik Jena, Germany) following the manufacturer’s instructions and quantified with a NanoDrop1000 Spectrophotometer (Thermo Scientific, Bonn, Germany). 150 ng extracted RNA was subjected to cDNA synthesis using a SensiFAST™ cDNA synthesis kit (Bioline GmbH, Germany) at 25 °C for 10 min and 42 °C for 15 min, 85 °C for 5 min. Gene expression levels of specific mRNAs were quantified by real-time PCR using the SensiFAST™ SYBR® No-ROX Kit on a Light Cycler 96 instrument (Roche). PCR products of each gene were initially cloned in the pGEM-T vector (Promega) and sequenced to verify the specificity of the primer pairs before analysis. Five different dilutions of cloned plasmids were used as standards and amplified with cDNA samples in each run. PCR amplicon was verified for each run using melting curve analysis and agarose gel electrophoresis of PCR products (Supplementary Fig. 3). RPLP0 served as an internal reference for gene expression analysis. Primer sequences were presented in Supplementary Table 1.

### Capillary western analysis

Automated Capillary Western was used to detect the target protein expression. After 6-day culture, GCs were washed using cold 1 × PBS and harvested by MPER protein extraction buffer (Thermo Fisher Scientific) with protease and phosphatase inhibitor cocktails. Protein samples were obtained by centrifugation at 12,000×*g* for 3 min at 4 °C, and the concentration of the protein supernatant was determined using a Micro BCA Protein Assay kit (23235;Thermo Scientific Pierce Biotechnology). Capillary electrophoresis were performed using the Wes system (ProteinSimple, Bio-Techne, USA) according to the manufacturer’s protocol. Protein samples were mixed with Wes sample master mix and heat denatured, then loaded onto the assay plate with other components which include primary antibodies (Supplementary Table 2), chemiluminescence substrate, blocking reagent, wash buffers, and secondary antibodies (anti-mouse DM-002 and anti-rabbit DM-001, ProteinSimple) in appropriate wells. The assay plate was loaded onto the Wes instrument for electrophoresis processing and analysis.

### Data and statistical analysis

Statistical analyses were carried out using the GraphPad Prism 10.1.1 licensed software (www.graphpad.com). The glucose data values in Fig. 1B and ECAR data in Fig. 1E were analyzed using two-way ANOVA. The remaining data were analyzed using one-way ANOVA. Unpaired t tests were performed for analyzing the SLC2A10 knockdown and BSA versus OA + PA + SA- treated samples. The statistical significance was expressed as one to four stars (*p < 0.05; **p < 0.01; ***p < 0.001; ****p < 0.0001).

## Results

### UFAs induce aerobic glycolysis

The collection and culture of primary GCs are schematically represented in Fig. 1A. To determine the effect of fatty acids on glucose consumption, we treated GCs with OA, PA, SA, and ALA at four different concentrations: 50 μM, 100 μM, 200 μM, and 400 μM while corresponding levels of BSA were used as vehicle control for each fatty acid concentration, and analyzed the glucose levels in the conditioned media. Results revealed that supplementation of the UFAs, OA and ALA caused significantly lower glucose levels in spent media compared to BSA (vehicle controls), PA and SA treatments (Fig. 1B), indicating an increased glucose consumption in UFA treated GCs. In contrast, saturated fatty acids PA and SA did not cause significant differences in glucose levels compared to BSA. We then wondered whether the glucose consumed under the influence of UFAs was used for aerobic glycolysis. We, therefore, assayed lactate levels in conditioned media after treating cells with 200 µM of different NEFAs as this particular concentration showed a significant impact on glucose utilization and also in close proximity to the physiological range of various fatty acids in bovine follicular fluid. Results indicate that OA and ALA but not PA and SA treatments significantly induced lactate production compared to the BSA treatment (Fig. 1C), indicating a higher degree of aerobic glycolysis in UFA treated GCs.

To clarify further that the increased glucose consumption in UFA treated cells is accompanied by an increased glycolytic rate, we measured ECAR using a seahorse XFe96 analyzer. After culturing GCs with OA, ALA and BSA for six days and after starvation for 1 h, addition of glucose instantaneously induced ECAR in all the treatments (Fig. 1D, the blue column) with ALA showing significantly higher glycolysis compared BSA (Fig. 1E). Addition of oligomycin resulted in a sharp induction of ECAR in OA and ALA treated cells compared to BSA control (Fig. 1D, the green column). Oligomycin is a mitochondrial ATP synthase inhibitor, which blocks proton conductance across the mitochondrial ATP synthase complex and inhibits the synthesis of ATP^41,42^. Glycolytic capacity and glycolytic reserve of GCs were found to be significantly higher after treatment with OA and ALA compared to BSA (Fig. 1E). A small but significant increase in non-glycolytic acidification was also observed in UFA treated cells from the ECAR readings before to glucose addition (Fig. 1D,E). ECAR was decreased rapidly after the addition of 2DG in all groups. 2DG is a glucolse analog and is converted into 2-deoxyglucose-6-phosphate (2DG-6P) by hexokinase upon uptake. 2DG-6P cannot be used by phosphoglucose isomerase in the next enzymatic step of glycolysis and it accumulates as a metabolic dead end^43^. We next verified the induction of glucose uptake using 2-NBDG, a fluorescent glucose analog, using a flow cytometer. Results showed a substantial increase in the accumulation of 2-NBDG by OA compared to BSA (Fig. 1F,G). While SA did not cause any significant differences, interestingly, PA treatment caused a significant decrease in 2-NBDG accumulation compared to BSA.

### Mitochondrial respiration and ATP production

**Figure 2:**
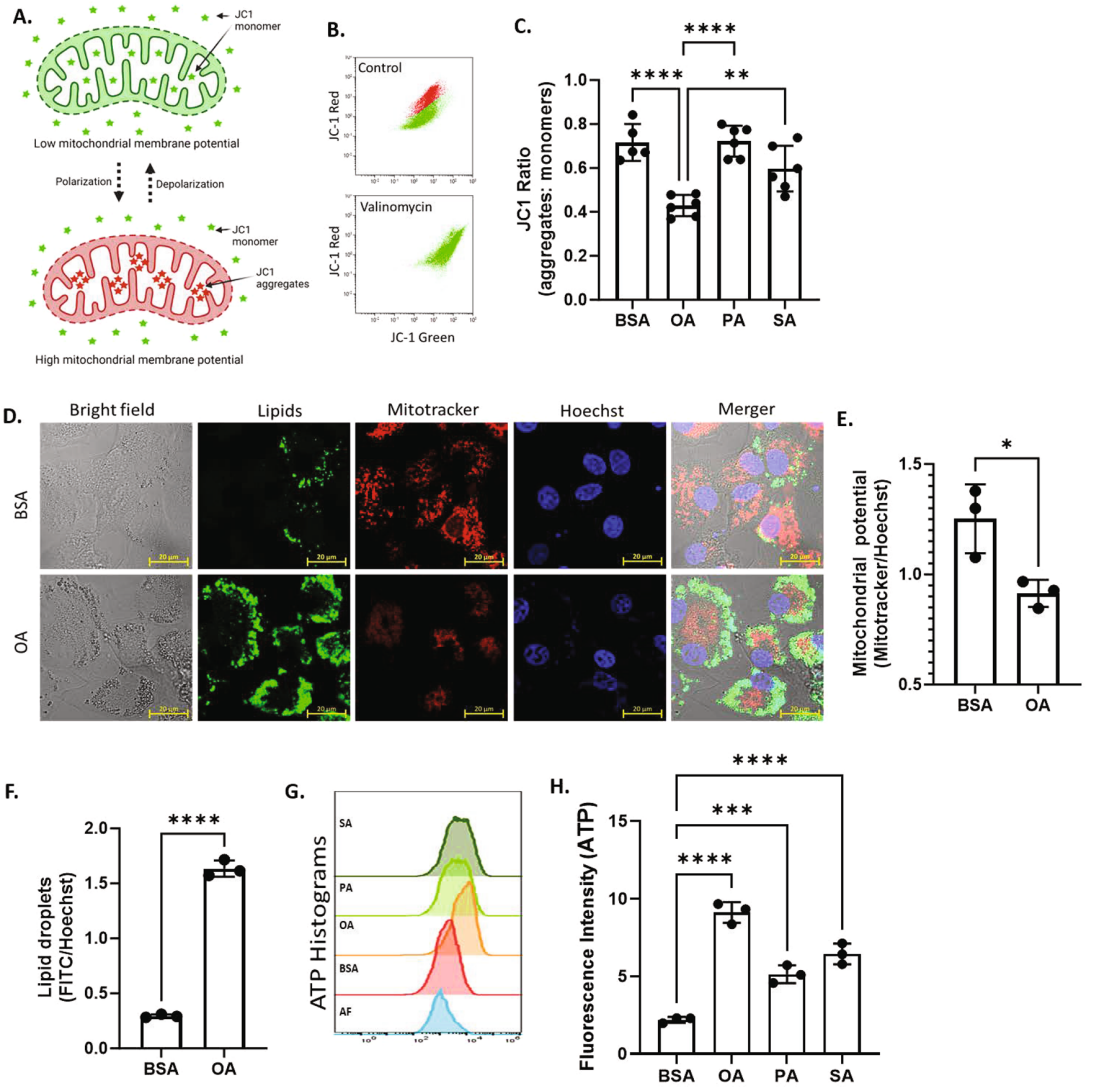
UFAs impair the mitochondrial membrane potential. (**A**) Schematic representation of JC1 accumulation under low and high mitochondrial membrane potential (MMP) states. Cells with low MMP accumulate JC-1 in monomeric form and show green fluorescence, while cells with high MMP contain JC1-aggregates showing red fluorescence. (**B**) Validation of JC-1 staining using valinomycin, a mitochondrial depolarizing agent, in flowcytometry. In valinomycin treated cells, JC1 is accumulated in monomeric form compared to the control cells. (**C**) Quantification of MMP, represented as JC-1 aggregates: monomer ratio in different fatty acid treated cells (n = 6). (**D**) Representative fluorescence microscopy images of GCs stained for lipid droplets (Lipids, green), mitochondria (Mitotracker, red), and nuclei (Hoechst, blue) in BSA and OA treated cells. (**E**) Quantification of MMP in BSA and OA treated cells from the fluorescence microscopy images (n = 3). (**F**) Quantification of lipid content in BSA and OA treated cells from the fluorescence microscopy images (n = 3). (**G**) Representative flowjo histograms of ATP (Adenosine triphosphate) levels in flowcytometer. (**H**) Quantification of ATP levels in cells treated with different fatty acids (n = 3). Probability values < 0.05 were considered statistically significant and are designated with up to four asterisk symbols to inform the strength of significant difference (*p < 0.05; **p < 0.01; ***p < 0.001, ****p < 0.0001). n = indicates the number of independent cell culture replicates analyzed. “AF” represents autofluorescence.

Increased glucose consumption and lactate production in UFA treated cells suggests the metabolic shift towards aerobic glycolysis. To verify this, we measured the mitochondrial membrane potential (MMP) using JC-1 staining after culturing cells with different fatty acids. Under low MMP, JC-1 maintains its monomeric form and shows green fluorescence. On the contrary, JC-1 monomers aggregate and emit red fluorescence at high MMP (Fig. 2A). The change in MMP was measured as JC-1 aggregates:monomer ratio. The JC-1 staining was initially tested in GCs using valinomycin, a mitochondrial membrane-depolarizing agent. Valinomycin treated cells emitted only green fluorescence compared to control cells as shown in flow cytometer histograms in Fig. 2B. We observed a significant decrease in JC-1 aggregates: monomer ratio in OA-treated GCs compared with BSA treatment, while there were no significant changes after PA and SA treatment (Fig. 2C), indicating that OA reduced MMP of GCs while inducing the glycolysis. As this is an important observation, we further validated the differences in MMP in BSA and OA treated cells using a voltage sensitive fluorescence mitotracker staining. As shown in Fig. 2D–F, BSA treated cells showed low lipid droplet content but high MMP. On the contrary, OA treated cells showed increased lipid droplet accumulation but low MMP, which is in line with the earlier JC1 staining. It is well known that MMP is positively related to ATP production. We therefore examined ATP levels in GCs and found increased intracellular ATP levels in all fatty acids treatments compared to BSA (Fig. 2G,H). Surprisingly, despite decreased MMP, OA caused the highest rise in ATP levels compared to BSA. The rise was also significantly higher compared to PA, and SA treated cells.

### UFAs upregulate SLC2A10 expression in GCs

**Figure 3:**
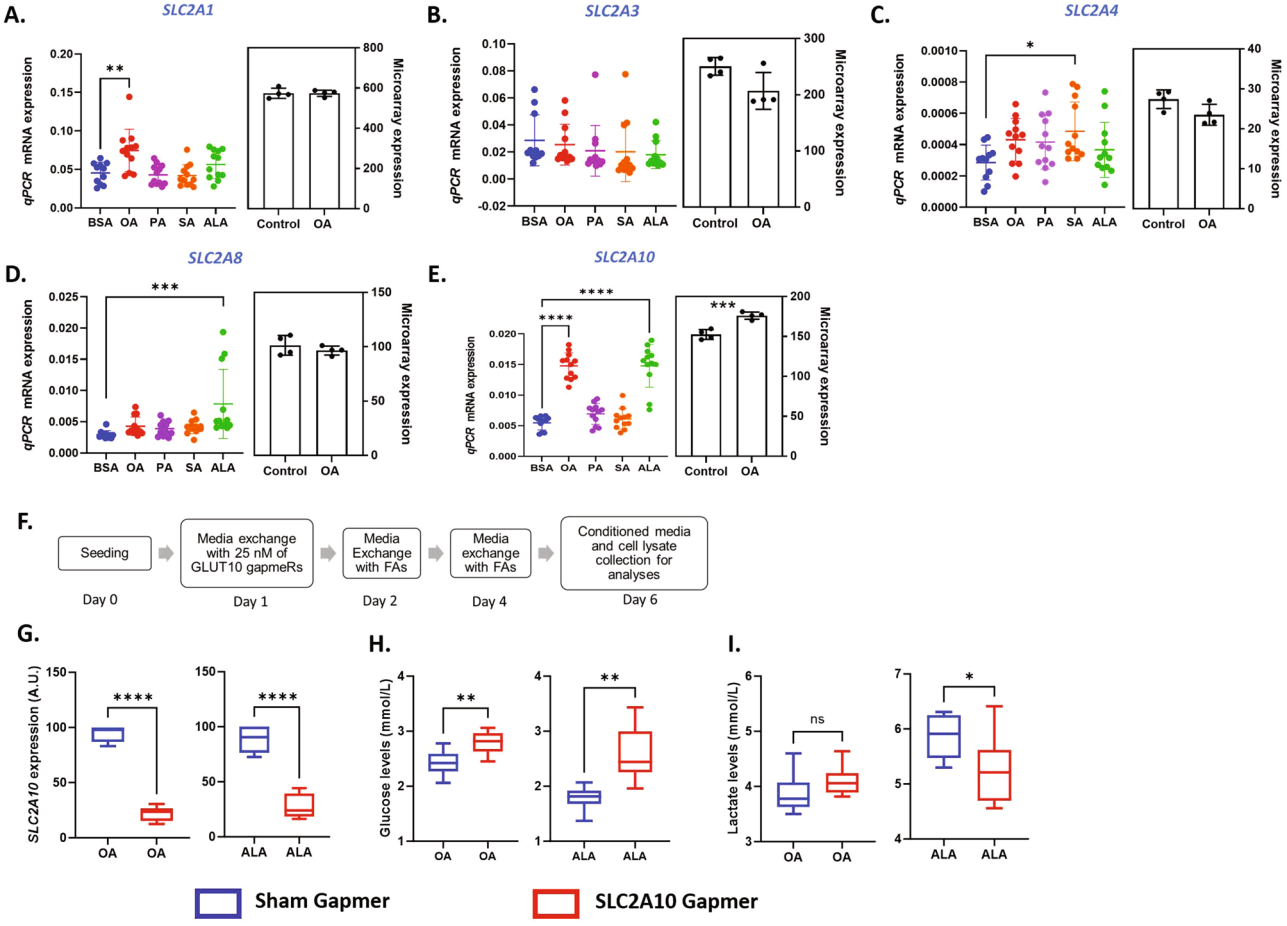
GLUT10 involved in UFA induced glucose uptake. (**A**) mRNA abundance data of SLC2A1 (n = 12) in qPCR analysis normalized to RPLP0 (left), and microarray analysis in inset (n = 4). (**B**) mRNA abundance data of SLC2A3 (n = 12) in qPCR analysis normalized to RPLP0 (left), and microarray analysis in inset (n = 4). (**C**) mRNA abundance data of SLC2A4 (n = 12) in qPCR analysis normalized to RPLP0 (left), and microarray analysis in inset (n = 4). (**D**) mRNA abundance data of SLC2A8 (n = 12) in qPCR analysis normalized to RPLP0 (left), and microarray analysis in inset (n = 4). (**E**) mRNA abundance data of SLC2A10 (n = 12) in qPCR analysis normalized to RPLP0 (left), and microarray analysis in inset (n = 4). (**F**) Schematic representation of the GLUT10 knockdown procedure. (**G**) Normalized SLC2A10 mRNA expression (A.U.) after the knockdown (kd) in OA-treated GCs (left) and ALA-treated GCs (right) (n = 8–9). (**H**) Glucose levels in spent media of OA-treated GCs (left) and ALA-treated GCs (right) upon knockdown (n = 9). (**I**) Lactate levels in spent media of OA-treated GCs (left) and ALA-treated GCs (right) upon knockdown (n = 9). Probability values < 0.05 were considered statistically significant and are designated with up to four asterisk symbols to inform the strength of significant difference (*p < 0.05; **p < 0.01; ***p < 0.001, ****p < 0.0001). n = indicates the number of independent cell culture replicates analyzed.

We then asked whether UFAs induced glucose transport involves the upregulation of glucose transporters in GCs. For this, we took advantage of the previously published transcriptome data in OA treated GCs^44^, which showed that only GLUT10 encoding SLC2A10 was significantly upregulated by OA compared to control amongst different glucose transporter genes. We analyzed mRNA abundances of the five most highly expressed GLUTs (SLC2A1, SLC2A3, SLC2A4, SLC2A8 and SLC2A10) by qPCR upon treating GCs with different fatty acids (Fig. 3). The qPCR data of these genes were compared with the published OA transcriptome data. Results indicated that SLC2A1 expression was increased by OA treatment but not by other fatty acids in qPCR, whereas transcriptome data indicated no induction of SLC2A1 by OA (Fig. 3A). SLC2A3 and SLC2A4 expressions were not regulated by UFAs, shown both by qPCR and transcriptome analyses (Fig. 3B,C). However, SLC2A4 expression was increased by SA in qPCR analysis (Fig. 3C). SLC2A8 was induced by ALA in qPCR but not by other fatty acids (Fig. 3D). Importantly, SLC2A10 expression was found to be significantly induced by both OA and ALA in qPCR analysis and also by OA in the referred transcriptome data (Fig. 3E). In contrast, PA and SA failed to induce SLC2A10 expression. This suggests that GLUT10 may be a potential glucose transporter involved in glucose uptake by UFAs in ovarian granulosa cells.

### GLUT10 mediates UFA-driven glucose uptake in GCs

Since UFAs dramatically induced the expression of SLC2A10, which was not very well annotated for its glucose uptake functions previously, we performed SLC2A10 gene knockdown in cultured GCs using the antisense GapmeR technology and examined the glucose and lactate levels in the spent media of OA and ALA treated GCs (Fig. 3F). A significant reduction of SLC2A10 expression following antisense GapmeR treatment indicated the efficacy of the SLC2A10 gene silencing (Fig. 3G). Analyses showed that glucose levels in the spent media was significantly increased both in OA and ALA treated cells after the knockdown compared to sham control (Fig. 3H), indicating that the glucose uptake was reduced and indeed mediated by GLUT10 in UFA treated GCs. Lactate levels were also significantly decreased in ALA treated cells after the knockdown (Fig. 3I) but not in OA treatment.

## AKT and ERK phosphorylation regulate SLC2A10 expression

**Figure 4:**
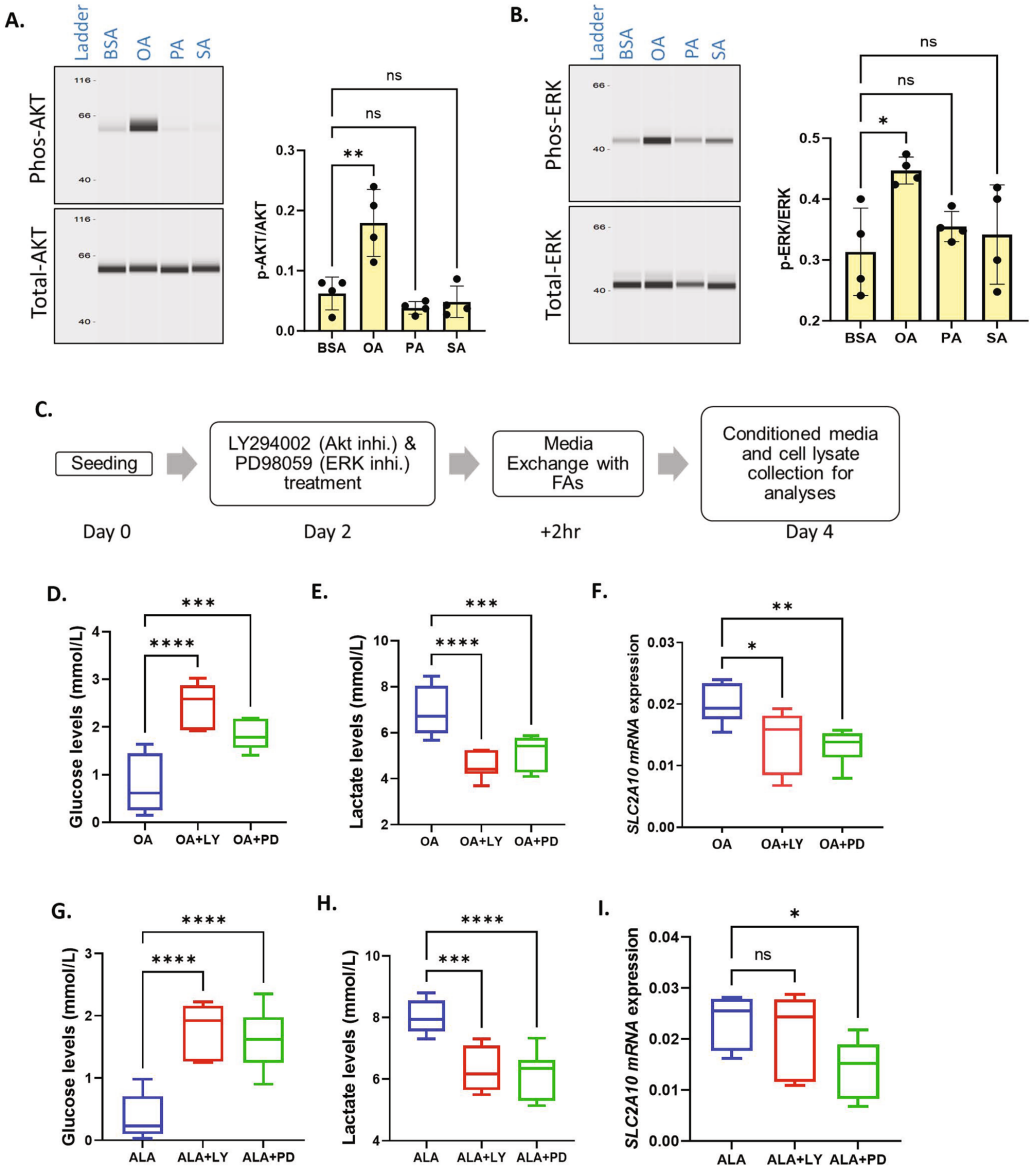
UFAs regulate glucose uptake by activating AKT and ERK signaling. (**A**) Digitally constructed capillary western analysis images of total AKT and phosphor-AKT intensities in BSA, OA, PA and SA treated cells and their quantification (n = 4). The image and quantification is obtained using high dynamic range exposure procedure in simple western instrument. (**B**) Digitally constructed western images of total ERK and phosphor-ERK intensities in BSA, OA, PA and SA treated cells and their quantification (n = 4). The image and quantification is obtained using high dynamic range exposure procedure in simple western instrument. (**C**) Schematic representations of the AKT inhibitor (LY294002) and ERK inhibitor (PD98059) treatment procedure. (**D**) Glucose levels in culture media in the presence or absence of AKT (LY) and ERK (PD) inhibitors in OA-treated cells (n = 8). (**E**) Lactate levels after inhibition of AKT and ERK signaling in OA-treated cells (n = 8). (**F**) SLC2A10 mRNA levels after inhibition of AKT and ERK signaling in OA-treated cells (n = 9). (**G**) Glucose levels after the inhibition of AKT and ERK signaling in ALA-treated cells (n = 9). ( H ) Lactate levels after the inhibition of AKT and ERK signaling in ALA-treated cells (n = 9). ( I ) SLC2A10 mRNA levels after the inhibition of AKT and ERK signaling in ALA-treated cells (n = 9). Probability values < 0.05 were considered as statistically significant and are designated with up to four asterisk symbols to inform the strength of significant difference (*p < 0.05; **p < 0.01; ***p < 0.001, ****p < 0.0001). ‘n’ represents the number of independent cell culture replicates analyzed.

Unsaturated fatty acids have been reported to induce the phosphorylation of AKT and ERK in various cell types^45–47^. Similarly, pathway analysis of transcriptome data in OA treated cells also indicated the activation of ERK and AKT signaling ^44^. Therefore, to determine whether UFAs induce these signaling pathways in GCs under the present long-term cell culture conditions, we analyzed AKT and ERK1/2 phosphorylation on day 6 of culture at which glucose measurement and other analyses were performed (Fig. 1A). Capillary western analysis showed that OA significantly induced phosphorylation of AKT and ERK proteins compared to BSA. In contrast, PA and SA did not induce the phosphorylation (Fig. 4A,B). To analyze whether these signaling pathways affect glucose uptake, lactate production and GLUT10 expression, we pretreated GCs with chemical inhibitors LY294002 and PD98059 (LY and PD) to specifically inhibit AKT and ERK signaling pathways, respectively, followed by UFA treatment (Fig. 4C). Results showed that the glucose levels in the spent media were significantly elevated upon inhibition of AKT and ERK phosphorylation (Fig. 4D,G), indicating a reduction in glucose uptake by the cells. In contrast, the lactate levels were markedly decreased in the spent media of inhibitor treated cells (Fig. 4E,H). This suggests that OA and ALA enhance glucose utilization by inducing AKT and ERK phosphorylation. Both AKT and ERK inhibitors inhibited the GLUT10 expression in OA treated cells, whereas only the ERK inhibitor has significantly inhibited the GLUT10 expression in ALA treated cells. AKT inhibitor has caused a nonsignificant decrease in SLC2A10 expression in ALA treated cells (Fig. 4F,I).

### PA and/or SA inhibit OA induced changes in the glucose metabolism

**Figure 5:**
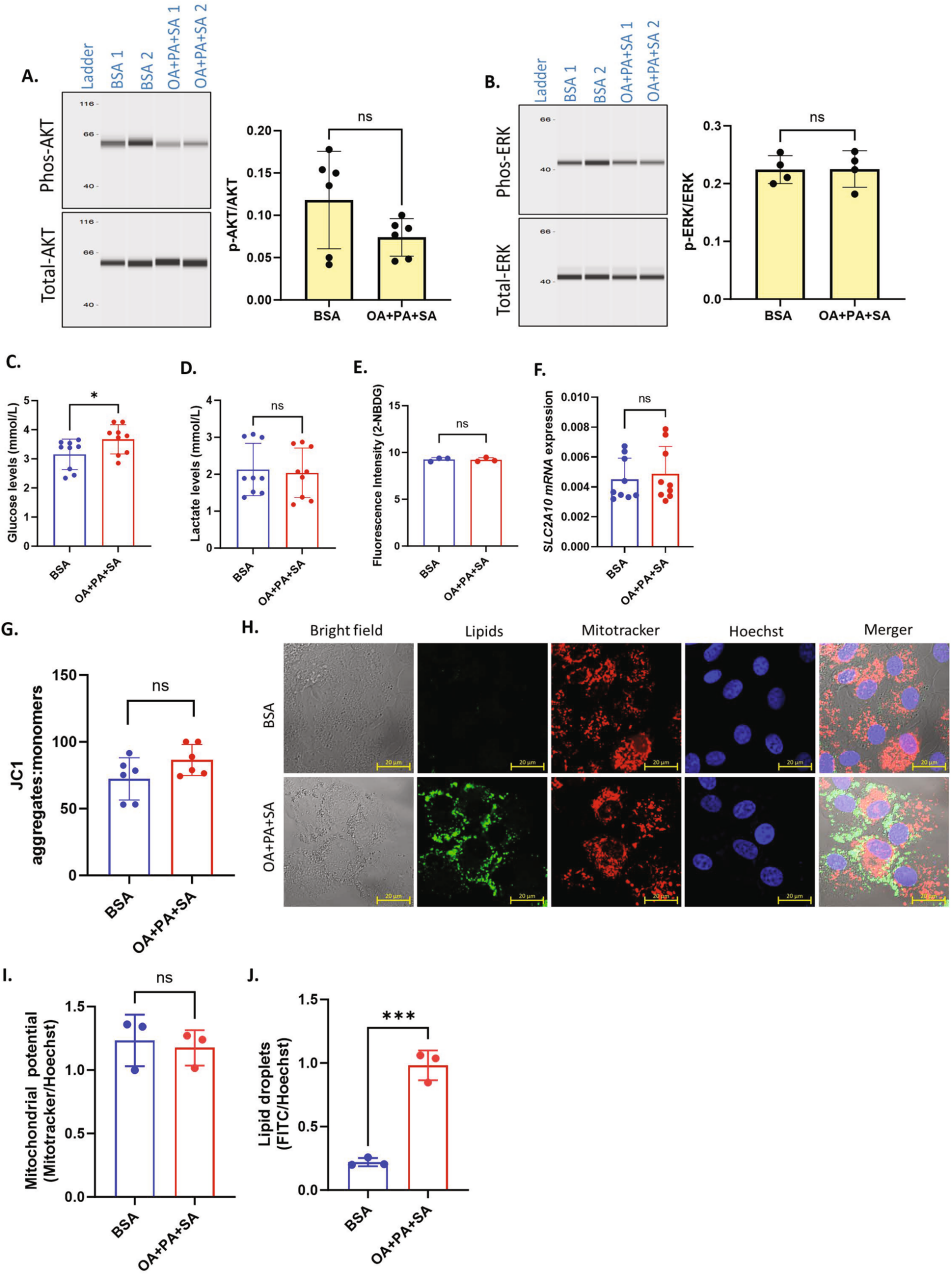
Physiological fatty acid mixes do not affect glucose utilization. (**A**) Digitally constructed western images of total AKT and phosphor-AKT intensities in BSA and OA 200 µM + PA100 µM + SA100 µM treated cells and their quantification (n = 6). The image and quantification is obtained using high dynamic range exposure procedure in simple western instrument. (**B**) Digitally constructed western images of total ERK and phosphor-ERK intensities in BSA and OA 200 µM + PA100 µM + SA100 µM treated cells and their quantification (n = 4). The image and quantification is obtained using high dynamic range exposure procedure in simple western instrument. (**C**) Glucose levels in the conditioned media after BSA and OA + PA + SA treatments (n = 9). (**D**) Lactate levels in the conditioned media after BSA and OA + PA + SA treatments (n = 9). (**E**) 2-NBDG uptake in BSA and OA + PA + SA treated cells (n = 3). (**F**) GLUT10 mRNA expression in BSA and OA + PA + SA treated cells (n = 9). (**G**) Quantification of mitochondrial membrane potential (MMP) using JC-1 in BSA and OA + PA + SA treated cells (n = 6). (**H**) Representative fluorescence microscopy images of GCs stained for lipid droplets (Lipids, green), mitochondria (mitotracker, red), and nuclei (Hoechst, blue) in BSA and OA + PA + SA treated cells. (**I**) Quantification of MMP in BSA and OA + PA + SA treated cells from the fluorescence microscopy images (n = 3). (**J**) Quantification of lipid droplets in BSA and OA + PA + SA treated cells from the fluorescence microscopy images (n = 3). Probability values < 0.05 were considered statistically significant and are designated with up to four asterisk symbols to inform the strength of significant difference (***p < 0.001). ‘n’ represents the number of independent cell culture replicates analyzed. “OA + PA + SA” represents cells treated with OA (200 µM) mixed with PA (100 µM) and SA (100 µM), “BSA” represents cells treated with bovine serum albumin (vehicle control).

**Figure 6:**
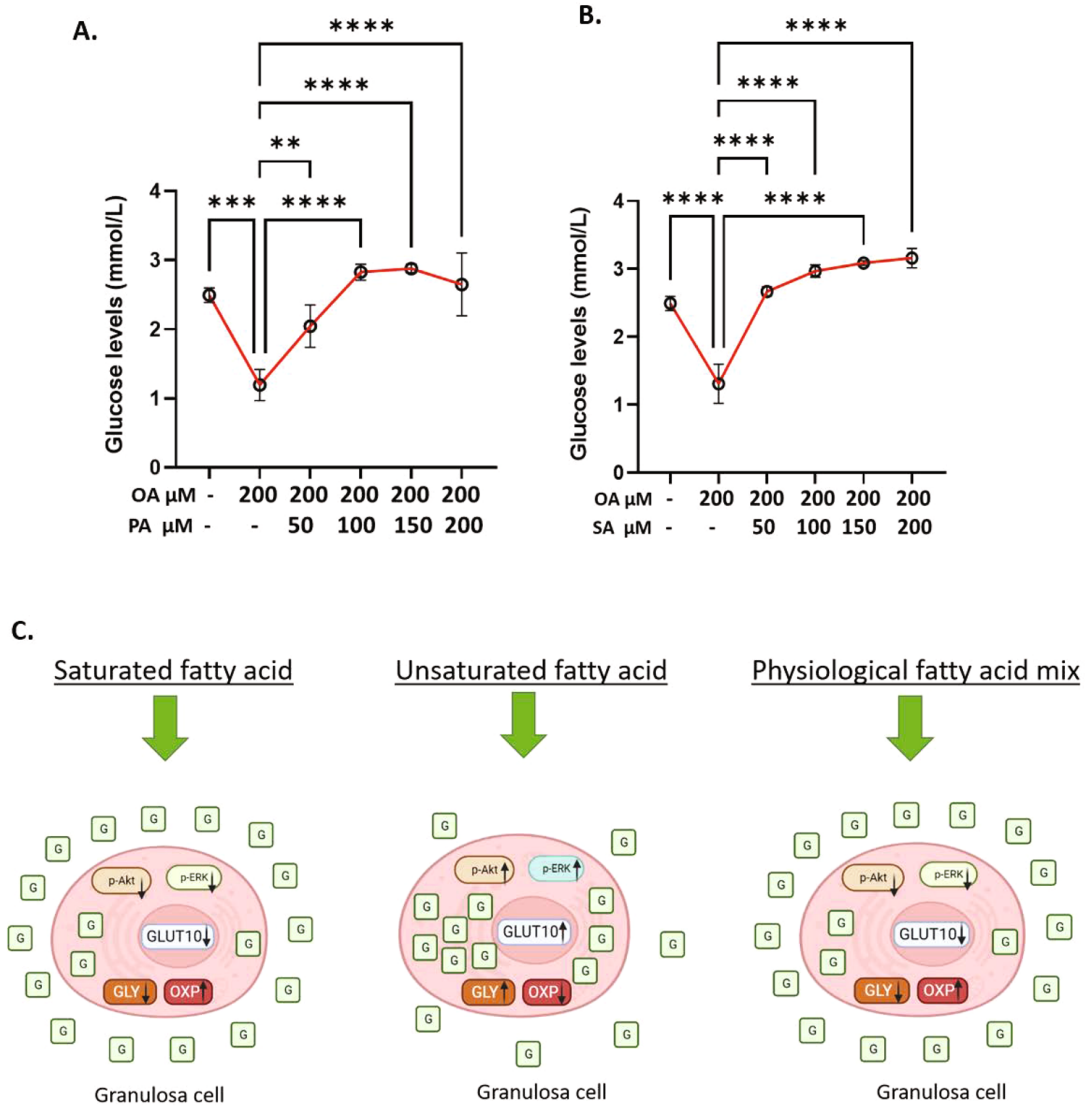
PA and SA may individually reverse UFA induced glucose consumption in GCs. (**A**) Glucose levels in granulosa cell conditioned media under different concentrations of PA in combination with 200 μM of OA (n = 4). (**B**) Glucose levels in granulosa cell conditioned media under different concentrations of SA in combination with 200 μM of OA (n = 4). Probability values < 0.05 were considered statistically significant and are designated with up to four asterisk symbols to inform the strength of significant difference (**p < 0.01; ***p < 0.001, ****p < 0.0001). ‘n’ represents the number of independent cell culture replicates analyzed. (**C**) The graphical representation of metabolic state and glucose consumption in granulosa cells in the presence of saturated fatty acid, unsaturated fatty acid and physiological fatty acid mixes. *G* glucose, *GLY* glycolytic pathway, *OXP* oxidative phosphorylation. ↑ increased, ↓ decreased.

Above experiments revealed that UFAs supplementation induced aerobic glycolytic metabolism by increasing glucose uptake via GLUT10, where as SFAs caused no significant changes in glucose uptake and mitochondrial respiration. We next wondered about the cumulative impact of UFAs and SFAs in GCs because both SFAs and UFAs mutually occur in all body fluids including ovarian follicular fluid. Since OA, PA and SA are the most abundant fatty acids in body fluids, we treated cells with a mix of OA (200 µM) + PA (100 µM) + SA (100 µM) to mimic the physiological concentration^48^ and analyzed glucose uptake and lactate production along with other parameters. At first, we analyzed AKT and ERK phosphorylation levels, which were earlier found to be induced by OA treatment (see Fige. 4A and B). Results showed that co-supplementation of PA and SA along with OA did not change AKT or ERK phosphorylation levels compared to BSA (Fig. 5A,B), indicating OA-induced phosphorylation of AKT and ERK was inhibited by the presence of SFAs in GCs. Unlike UFA treated cells, glucose levels in the spent media were significantly increased upon fatty acid mix treatment compared to the control, and no changes in lactate levels were observed between the groups (Fig. 5C,D). Further, analysis of 2-NBDG uptake and GLUT10 mRNA expression also revealed no significant changes compared to their corresponding controls (Fig. 5E,F). We then analyzed the MMP using JC1 (Fig. 5G) and MitoTracker™ Orange reagent (Fig. 5H,I). Both assays revealed no significant changes in the MMP of cells treated with fatty acid mixtures compared to the control. Fluorescence images and corresponding signal quantifications clearly showed increased lipid droplet accumulation in cells treated with fatty acid mixture (Fig. 5H,J). These data indicated that the presence of SFAs, PA and SA, prevented UFAs induced glucose uptake and rescued the mitochondrial metabolism in GCs. To further understand the potential of SFAs on inhibiting UFA driven glucose uptake, we performed biological titration-like experiments by supplementing cells with 50 μM, 100 μM, 150 μM, and 200 μM of PA or SA in combination with 200 μM of OA (Fig. 6A,B). Results showed a nearly complete inhibition of OA-induced glucose uptake upon PA or SA co-treatment at all concentrations, indicating that SFAs potently prevent the OA induced glucose consumption and glycolytic metabolism in GCs even at a 1/4th concentration to that of UFAs in the extracellular environment. Similar counter effects on glucose were observed in ALA-treated cells when co-treated with SA and PA (Supplementary Fig. 4). Overall, above data from OA + PA + SA treatments suggest that SFAs play a determining role by preventing the OA induced glucose uptake in GCs.

## Discussion

Glucose uptake by cells depends upon the metabolic needs and availability of glucose and is regulated by various endocrine, paracrine, and autocrine factors in different cells. It has been shown that disturbances in fatty acid metabolism could affect glucose homeostasis and cause moderate to severe health conditions, including insulin resistance in humans^49,50^. In the present study, analysis of glucose utilization in ovarian GCs upon supplementing OA, ALA, PA, and SA clearly indicated that UFAs induce glucose uptake in GCs (Fig. 1). In accordance with the present findings, a previous study reported that supplementation with various UFAs, including OA and ALA, led to increased glucose consumption in 3T3-L1 adipocytes, while no such effect was observed with SFAs^51^. In another study, treating rat adipocytes with PA caused insulin resistance and prevented glucose uptake^52^. Contrarily, PA has also been shown to induce glucose uptake in rat skeletal muscle cells over-expressed with GLUT4^53^. Similarly, SA has also induced glucose uptake via inhibiting protein tyrosine phosphatase 1b, thereby increasing insulin receptor signaling in T3-L1-GLUT4myc adipocytes^54^. Therefore, it appears that both SFAs and UFAs can induce glucose accumulation in different cells.

Most of the earlier studies analyzing the glucose uptake under fatty acid treatments did not follow the metabolic fate of glucose after the uptake and did not address the physiological scenario as both SFAs and UFAs are present together in all body fluids. We observed that the glucose transported from the media into UFAs-treated GCs was directed towards aerobic glycolysis, yielding lactate and increasing ECAR. Interestingly, cells have undergone aerobic glycolysis even though they can generate more energy via the electron transport chain under the given normoxic culture conditions (21% O_2_). This is plausibly because of the decreased mitochondrial activity (lower MMP) upon OA treatment leaving the cells no choice but undergo aerobic glycolysis for energy production. Surprisingly, despite low MMP, ATP levels were increased in OA treated cells compared to other treatments (Fig. 2). On the other hand, PA and SA treatments have also increased ATP levels compared to BSA control but the magnitute of increase was less than that of OA treated cells (Fig. 2H). Given the fact that glycolysis is a preferred metabolic pathway in GCs and the UFAs are not the favorite substrates for mitochondrial oxidation, UFA treated cells might turn to aerobic glycolysis to yield such a high level of ATP production. This ability might help the cells survive under very low oxygen pressure conditions in the non-vascularized follicular granulosa cell layer in the ovary^55^.

We found increased expression of SLC2A10 in OA and ALA, but not in PA and SA treated cells (Fig. 3E), suggesting that GLUT10 may be involved in UFA induced glucose uptake in GCs. Earlier studies on GLUT10 have mainly focused on Arterial tortuous syndrome (ATS), which is caused by loss of function mutations in SLC2A10^56^. In addition, GLUT10 was found to be a critical factor in maintaining the ascorbic acid levels necessary for adipogenesis. GLUT10 is being considered as a potential susceptibility locus for high-fat diet-induced type 2 diabetes mellitus^57^. Shawna and coworkers showed that GLUT10 mediates chronic activity-induced glucose uptake in mouse muscle^23^. Another study reported that SLC2A10 could be a target of lncRNA H22954, which inhibited GLUT10 expression and glucose uptake in leukemia cells^58^. Nevertheless, the function of GLUT10 still largely remains unknown. Therefore, we found GLUT10 as an interesting target for functional analysis. Silencing the SLC2A10 mRNA expression has significantly decreased the glucose uptake in UFA treated cells, indicating that GLUT10 directly contributes to glucose uptake in GCs. Although we could not verify the protein levels due to a lack of bovine reactive antibodies for GLUT10, a reduction of almost 80% of SLC2A10 mRNA indicates a clear silencing of gene expression after the GapmeR treatment. It is known that AKT and ERK signaling contribute to glucose uptake and metabolism in various cell types. Inhibition of AKT and ERK signaling prevented SLC2A10 induction, glucose uptake and lactate production in present UFA treated GCs. Interestingly, Jing Pu et al. reported that, contrary to the present observations, PA induced glucose uptake in skeletal muscle cells occurs via AKT and ERK1/2 pathways^53^. In our study, PA did not significantly affect AKT and ERK signaling, but the induction of these pathways by UFAs and their involvement in glucose uptake suggests a central role of AKT and ERK pathways in glucose uptake in various cells.

Under any physiological condition, both SFAs and UFAs mutually occur in body fluids. Therefore, the net effect of fatty acids on glucose uptake under physiological conditions could be better determined by treating cells with fatty acid mixes rather than deriving the impacts of individual fatty acids. Since OA, PA, and SA are abundant fatty acids in the body fluids, we have treated GCs with a mixture of these fatty acids (200 µM of OA + 100 µM of PA + 100 µM of SA) in a physiological ratio^48^ and analyzed nearly all parameters that were performed for individual fatty acid treatments (Fig. 5). Interestingly, presence of PA and SA prevented OA induced glucose utilization, lactate production and the upregulation of SLC2A10 expression, indicating that SFAs abolish the glucose uptake and its metabolism induced by UFAs in GCs. Most of the earlier studies were performed to analyze the effects of individual fatty acids and did not consider the impact of fatty acid mixes on glucose uptake in different cell types. A study in KGN and SVOG cells showed that PA supplementation caused insulin resistance by inhibiting glucose uptake and lactate production^37^, which is consistent with the present findings as SFAs inhibited the OA-induced glucose uptake and lactate production in bovine GCs. Marchut et al. demonstrated that SFAs (PA and SA) at a concentration of 1 mM added to the medium in the form of potassium salts inhibited both glucose utilization and lactate formation, with SA inhibiting glycolysis more strongly than PA (C16:0) in Ehrlich ascites tumor cells^59^. They further showed that the degree of inhibition depends on carbon chain length of SFAs with in the concentration range of 0.1–1.0 mM^59^. Kim and colleagues have demonstrated that elevated plasma NEFA levels following intralipid infusion in rats decreased insulin-stimulated glucose uptake by suppressing glycolysis in skeletal muscles^60^. From the present data, we infer that the presence of PA and SA in the infusion mixture might inhibit the AKT and ERK signaling induced by UFAs, thereby preventing glucose uptake upon intralipid infusions.

We conclude that UFAs induce glucose consumption and metabolism in GCs. However, the physiological NEFA mixes, containing both SFAs and UFAs, do not cause glucose consumption in GCs due to the presence of PA and SA, which inhibit UFA driven glucose utilization and cause a net neutral effect on metabolism (Fig. 6C). GLUT10 may play a key role in glucose uptake and metabolism in ovarian GCs upon UFAs supplementation. Since SLC2A10 silencing reduced glucose uptake in GCs, we suggest that GLUT10 could be explored as a potential novel target for glucose disorders in humans and animals.

### Supplementary Information

Supplementary Information.

## Data Availability

All representative data used to support presentfindings are included in the manuscript. Raw data sets can be available from the corresponding author upon request. The already published oleic acid transcriptome datasets referred in the manuscript can be found using the Gene Expression Omnibus accession number GSE152307.
