# Supplementary material for: Saturated fatty acids inhibit unsaturated fatty acid induced glucose uptake involving GLUT10 and aerobic glycolysis in bovine granulosa cells
Source: Sci Rep. 2024 Apr 30;14:9888. doi: 10.1038/s41598-024-59883-x (PMC11061182; doi:10.1038/s41598-024-59883-x)

Title:

Saturated fatty acids inhibit unsaturated fatty acid induced glucose uptake involving GLUT10 and aerobic glycolysis in bovine granulosa cells

Authors:

Xuelian Tao[1], Maryam Rahimi[2], Marten Michaelis[1], Solvig Görs[1], Julia Brenmoehl[1], Jens Vanselow[1] and Vijay Simha Baddela[1*]

Affiliations:

[1]Research Institute for Farm Animal Biology (FBN), 18196 Dummerstorf, Germany

[2]Abteilung Biotechnologie und Reproduktion landwirtschaftlicher Nutztiere, Georg-August-Universität Göttingen, 37037 Göttingen, Germany

***Corresponding author:**

Vijay Simha Baddela, Research Institute for Farm Animal Biology (FBN), Wilhelm-Stahl-Allee 2, 18196 Dummerstorf, Germany; Tel: +49 38208 68748; Fax: +49 38208 68752; e-mail: baddel@fbn-dummerstorf.de

Short title:

Fatty acid-induced glucose consumption and metabolism

Supplementary Table 1: PCR Primers

Gene	NCBI accession No.	Sequence	Size (bp)
RPLP0	NM_001012682	F: TGGTTACCCAACCGTCGCATCTGTA<br>R: CACAAAGGCAGATGGATCAGCCAAG	142
GLUT1	NM_174602.2	F: CATGACCATCGCGCTGGCGCTGC<br>R:AAGACGTAGGGTCCGCACAGTTGCTCC	252
GLUT3	NM_174603.3	F: GGCACCGAGGACGTGGCTCAGGAT<br>R: GCGCCGATAGTGGCATAGACCGGC	242
GLUT4	NM_174604.1	F: AGTCGCCTCCCCCTCGTCTACTGC<br>R: TGCCTCCCCAGCCAGGTCTCATTGT	245
GLUT8	NM_201528.1	F: CGGCGTCTGCGTCCTCACCAACTG<br>R: GGTCCAGCAAGGACGGGCAGTCAC	245
GLUT10	NM_001192439.3	F: GGCTTTGGACCCGTGACCTGGCTT<br>R: TGAAGCCCAGGCCGAAGACAGCAG	202

Supplementary Table 2: List of Antibodies

Antibody	Product No.	Brand	Source
Phospho ERK1/2	9101S	Cell Signaling	Rabbit
Total ERK1/2	4695S	Cell Signaling	Rabbit
Phospho Akt (Ser473)	9271S	Cell Signaling	Rabbit
Total Akt	9272S	Cell Signaling	Rabbit
Beta Actin	SC47778	Santa Cruz	Mouse

Supplementary Figure 1:

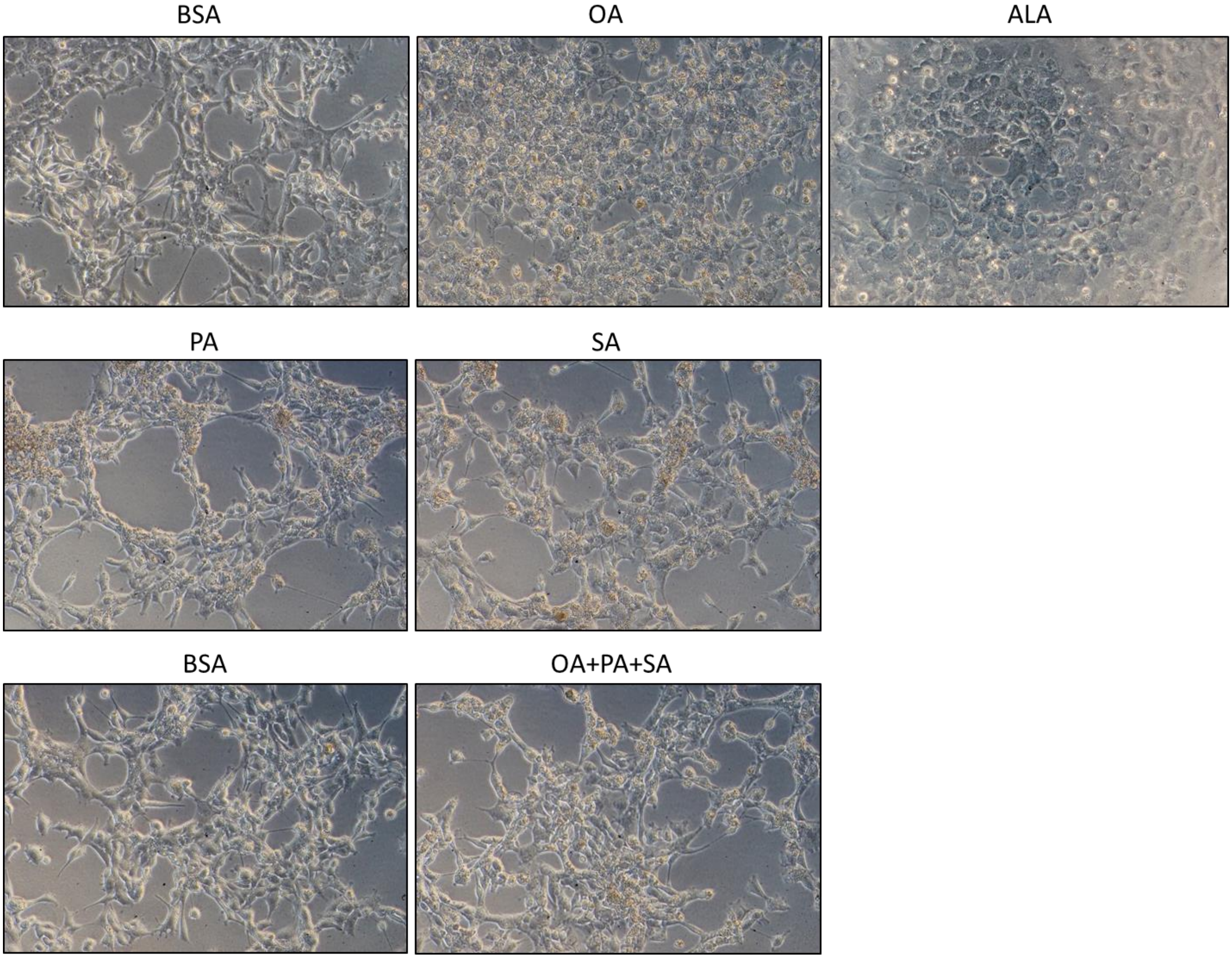

Supplementary Figure 1. Brightfield photomicrographs of cultured cells

Bovine granulosa cells were cultured with fatty acids as mentioned in Fig 1A in the manuscript. Photomicrographs of cultured granulosa cells were taken on day 6 with a Nikon microscope (20x).

Supplementary Figure 2:

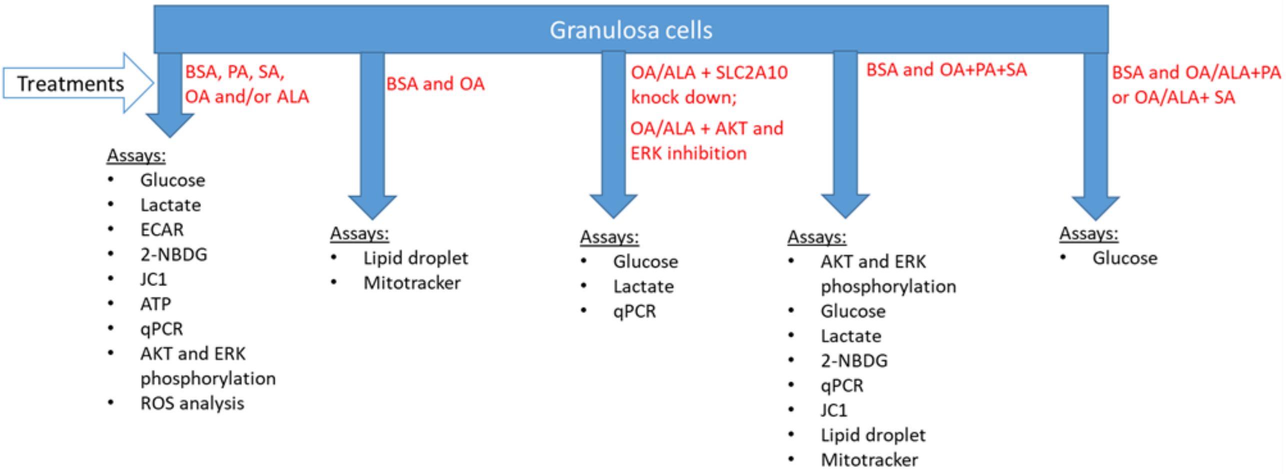

Supplementary Figure 2. Outline of all experimental treatments and techniques used in the present study

Supplementary Figure 3:

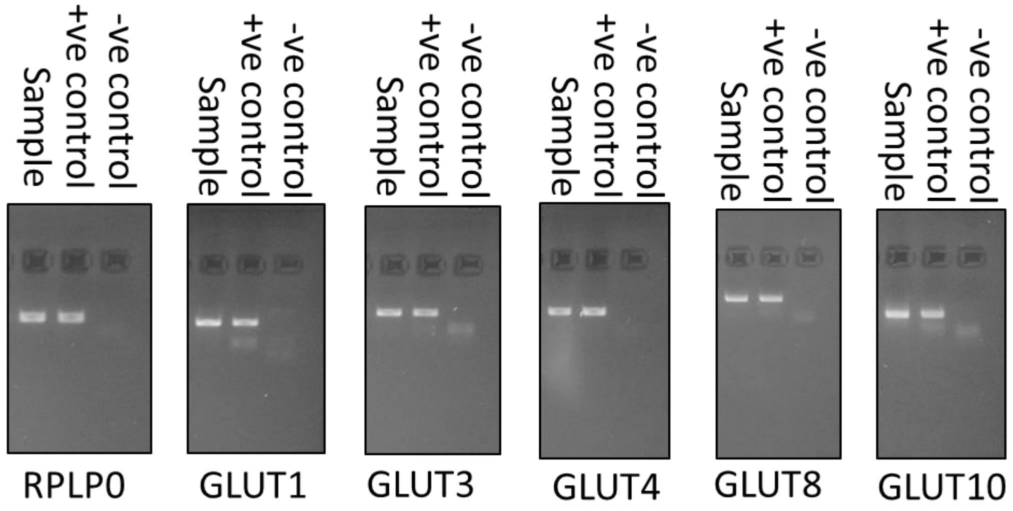

Supplementary Figure 3. Specificity of PCR products

Agarose gel electrophoresis indicates a specific and single amplified PCR product for the gene specific primer pairs used in the study. Positive control is the vector-cloned and sequence-verified template. Sample is the test sample; -ve control contains H_2O instead of DNA.

Supplementary Figure 4:

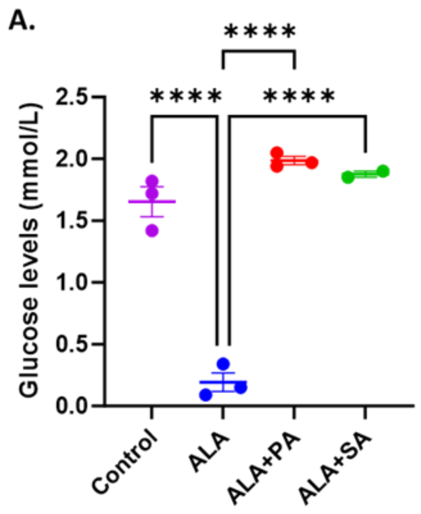

Supplementary Figure 4. SFAs prevent ALA-induced glucose uptake

(A) Glucose levels in conditioned media of BSA, 200 µM ALA, 200 µM ALA + 200 µM PA, and 200µM ALA + 200µM SA treated cells.

Each data point indicates an independent cell culture replicate. Probability values< 0.05 were considered statistically significant and are designated with up to four asterisk symbols to inform the strength of significant difference (* p<0.05; ** p<0.01; *** p<0.001, **** p<0.0001).

Supplementary Figure 5:

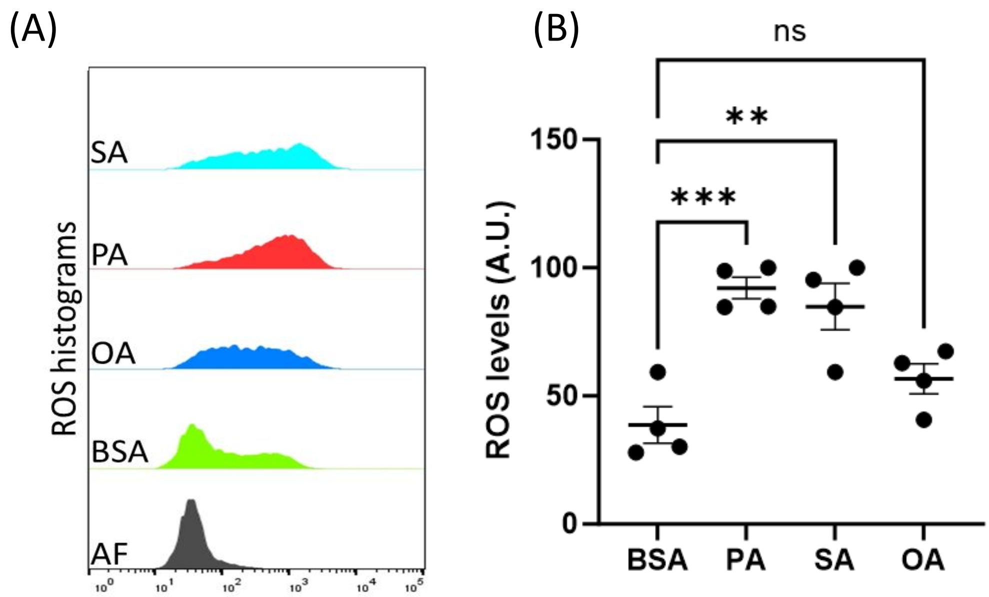

Supplementary Figure 5. Reactive oxygen species (ROS) quantification

(A) Representative flow cytometry histograms of ROS in GCs
(B) ROS levels in GCs treated with 200 µM different NEFA species and BSA.

Cell culture, detachment and washing steps was performed as depicted in manuscript section 2.5. The detached cells were incubated in DMEM containing 1 µM CellROX DeepRed (C10491, ThermoFisher Scientific) for 30 min at 37°C. After one wash step in DMEM, cells were analyzed by flow cytometry (Gallios, Beckman Coulter, USA) as per the manufacturer's instructions. Results indicate that ROS levels were increased by PA and SA compared to BSA. OA treatment did not affect the ROS levels. Each data point indicates an independent cell culture replicate. Probability values< 0.05 were considered statistically significant and are designated with up to four asterisk symbols to inform the strength of significant difference (* p<0.05; ** p<0.01; *** p<0.001, **** p<0.0001).

Full-length images of digitally constructed western lanes:

Figure 4a

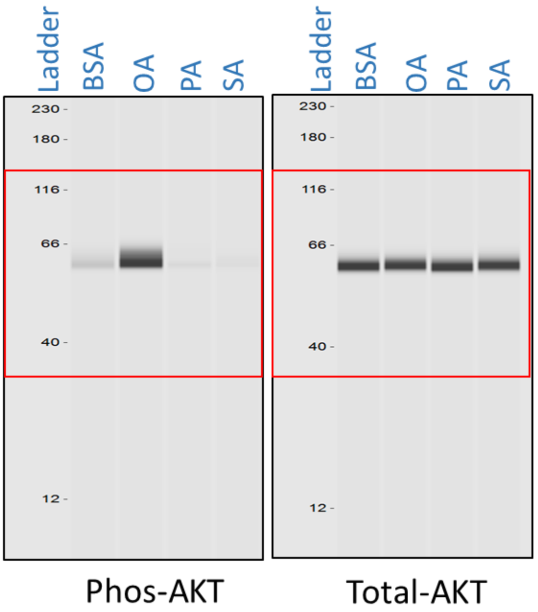

Figure 4b

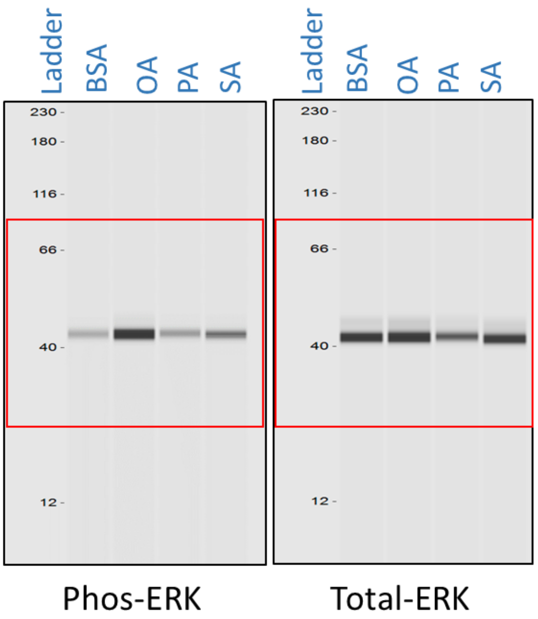

Figure 5a

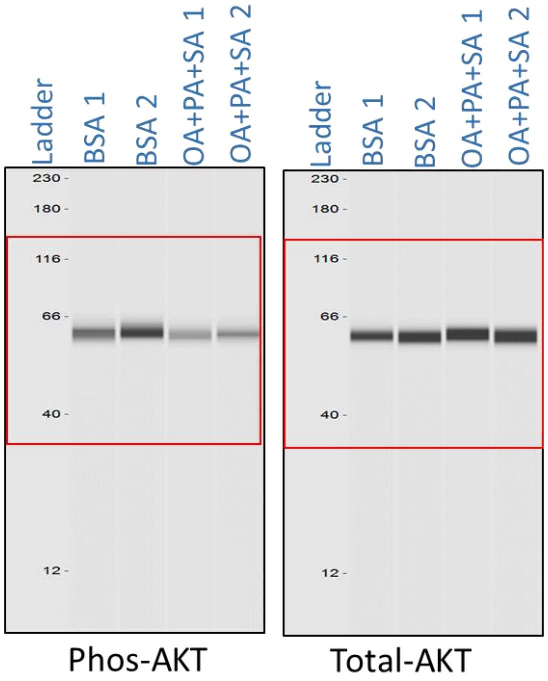

Figure 5b

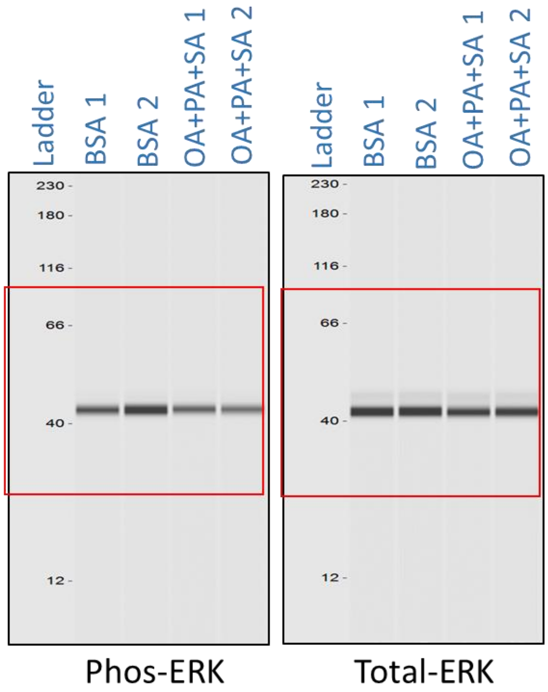

Supplement: Supplementary file 1 — Supplementary Information. [file 41598_2024_59883_MOESM1_ESM.pdf]
